# *In Vitro* and *In Vivo* Antibacterial Activities of a Novel Quinolone Compound, OPS-2071, against Clostridioides difficile

**DOI:** 10.1128/AAC.01170-20

**Published:** 2021-03-18

**Authors:** Daisuke Oka, Naomitsu Yamaya, Takuya Kuno, Yuta Asakawa, Toshiyuki Shiragiku, Liang Chen, Jingbo Xue, Abudusaimi Mamuti, Fangguo Ye, Jiangqin Sun, Kinue Ohguro, Hisashi Miyamoto, Yukitaka Uematsu, Katsuya Inagaki, Jie-Fei Cheng, Makoto Matsumoto

**Affiliations:** a Department of Medical Innovations, New Drug Research Division, Otsuka Pharmaceutical Co., Ltd., Tokushima, Japan; b Department of Drug Metabolism and Pharmacokinetics, Otsuka Pharmaceutical Co., Ltd., Tokushima, Japan; c Department of Investigative Toxicology, Nonclinical Research Center, Tokushima Research Institute, Otsuka Pharmaceutical Co., Ltd., Tokushima, Japan; d Department of Drug Safety Research, Nonclinical Research Center, Tokushima Research Institute, Otsuka Pharmaceutical Co., Ltd., Tokushima, Japan; e Otsuka Shanghai Research Institute, Shanghai, China; f Infectious Diseases Unit, Department of Medical Innovations, New Drug Research Division, Otsuka Pharmaceutical Co., Ltd., Tokushima, Japan; g Medicinal Chemistry Research Laboratories, New Drug Research Division, Otsuka Pharmaceutical Co., Ltd., Tokushima, Japan; h Pharmaceutical Business Division, Otsuka Pharmaceutical Co., Ltd., Tokyo, Japan

**Keywords:** OPS-2071, anti-*Clostridioides difficile* agent, new quinolone

## Abstract

OPS-2071 is a novel quinolone antibacterial agent characterized by low oral absorption that reduces the risk of adverse events typical of fluoroquinolone class antibiotics. The *in vitro* and *in vivo* antibacterial activities of OPS-2071 against Clostridioides difficile were evaluated in comparison to vancomycin and fidaxomicin. OPS-2071 exhibited potent antibacterial activity against 54 clinically isolated C. difficile strains with a MIC of 0.125 μg/ml (MIC_50_) and 0.5 μg/ml (MIC_90_), making it more active than vancomycin on a concentration basis (MIC_50_, 2 μg/ml; MIC_90_, 4 μg/ml) and comparable to fidaxomicin (MIC_50_, 0.063 μg/ml; MIC_90_, 8 μg/ml). OPS-2071 showed equally potent antibacterial activity against both hypervirulent and nonhypervirulent strains, while a significant difference in susceptibility to fidaxomicin was observed. Spontaneous resistance to OPS-2071 and vancomycin was not observed; however, resistance to fidaxomicin was observed at 4× MIC. The mutant prevention concentration of OPS-2071 was 16-fold lower than those of fidaxomicin and vancomycin, and the postantibiotic effect of OPS-2071 was longer than those of fidaxomicin and vancomycin. Also, OPS-2071 showed low systemic exposure, with OPS-2071 having 2.9% oral bioavailability at 1 mg/kg in rats. Furthermore, OPS-2071 showed significant *in vivo* efficacy at 0.0313 mg/kg/day (50% effective doses), 39.0-fold and 52.1-fold lower than those of vancomycin and fidaxomicin, respectively, in a hamster model of C. difficile infection. OPS-2071 has the potential to become a new therapeutic option for treating C. difficile infection.

## Abstract (precis)

OPS-2071 is a novel quinolone antibacterial agent characterized by low oral absorption that reduces the risk of adverse events typical of fluoroquinolone class antibiotics. The *in vitro* and *in vivo* antibacterial activities of OPS-2071 against Clostridioides difficile were evaluated in comparison to vancomycin and fidaxomicin.

## INTRODUCTION

Many commercially available antibiotics have the potential to cause diarrhea during treatment of infectious diseases (1), and they often disrupt the normal balance of intestinal flora. This can lead to an increase in pathogenic bacteria that may eventually induce so-called antibiotic-associated diarrhea (AAD). One of the most important causes of AAD is expansion of C. difficile, as it is resistant to many currently available antibiotics. It is specifically designated C. difficile infection (CDI) or C. difficile-associated diseases (CDAD), which are implicated in 10 to 25% of AAD and the most prevalent infective cause of AAD (2, 3). In 2011, the estimated incidence of CDI in the United States was 453,000 and the estimated annual mortality was 295,000 (4).

C. difficile is capable of producing three toxins, toxin A (TcdA), toxin B (TcdB), and C. difficile transferase toxin (CDT). These toxins are closely associated with certain clinical symptoms (5). In the early 2000s, hypervirulent strains such as the PCR ribotype 027, referred to as BI/NAP1/027, emerged and spread rapidly (6, 7). The BI/NAP1/027 strain produces all three of the toxins referred to above. They damage the gut barrier, leading to severe enterotoxicity in humans, and the BI/NAP1/027 strain is characterized by high-level fluoroquinolone resistance (8, 9).

Since the toxins of C. difficile damage the intestinal epithelial barrier and promote mucosal inflammation, CDI affects gut-related diseases. There are many reports describing how CDI dramatically increases in patients with inflammatory bowel disease (IBD). Consequently, morbidity, mortality, the need for surgery, and health care costs have been increasing due to CDI in IBD patients compared with IBD patients who are not infected (10–14). Therefore, there is general agreement that CDI is the most common gastrointestinal infection in patients with IBD. Treatment of CDI is a critical subject for gut-related diseases such as IBD.

Given the current range of treatment options, vancomycin and fidaxomicin are the preferred first-line therapeutic agents for the initial episode of CDI, but their use in cases of multiple recurrences is not well established. Metronidazole is also used to treat CDI but is only recommended for treatment of the initial episode in nonsevere cases (15). Over the preceding decade, new CDI treatments have been developed, such as fecal microbiota transplantation (FMT). FMT restores the diversity of the gut microbiota and has achieved cure rates exceeding 85%. However, there is a risk that the donor stool may cause infection in immunocompromised patients (16). While FMT is a promising therapy, it has not been widely accepted for broad clinical use. Thus, there is a pressing need for the development of a new therapeutic agent for CDI that can successfully address these issues.

**FIG 1:**
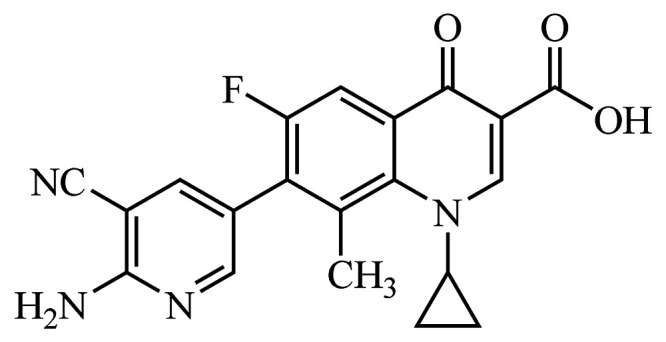
Chemical structure of OPS-2071.

OPS-2071, 7-(6-amino-5-cyanopyridin-3-yl)-1-cyclopropyl-6-fluoro-8-methyl-4-oxo-1,4-dihydroquinoline-3-carboxylic acid, is a novel quinolone antibacterial agent (Fig. 1) developed at Otsuka Pharmaceutical Co., Ltd., targeting intestinal infection pathogens, including C. difficile. It has decreased potential for absorption from the intestine, which in turn reduces the adverse events commonly associated with the fluoroquinolone class of antibiotics.

In order to assess the potential utility of OPS-2071 against CDI, the evaluation of *in vitro* and *in vivo* antibacterial activity, spontaneous resistance, and pharmacokinetics was performed and compared to reference compounds.

## RESULTS

### Antibacterial activity of OPS-2071 against 54 clinically isolated strains of C. difficile.

The *in vitro* antibacterial activities of OPS-2071 and other relevant compounds against 54 clinically isolated C. difficile strains obtained from Aino Hospital (Osaka, Japan) and Miroku Laboratory (Nagano, Japan) are shown in Table 1 and Table S1 in the supplemental material. As expected, commonly used quinolone antibiotics such as ciprofloxacin and levofloxacin are inactive against C. difficile isolates. Compared with conventional therapeutic agents (vancomycin, metronidazole, and fidaxomicin), the MIC_50_ and MIC_90_ of OPS-2071 were, respectively, 16-fold and 8-fold lower than those of vancomycin and metronidazole and 2-fold higher and 16-fold lower than that of fidaxomicin. Using the method described by the Clinical and Laboratory Standards Institute (CLSI), the MICs of OPS-2071, vancomycin, and fidaxomicin against a quality control strain, C. difficile ATCC 700057, were 0.031, 2, and 0.063 μg/ml, respectively (17). These results indicate that OPS-2071 possesses comparable activity to that of fidaxomicin and was more active than both vancomycin and metronidazole against C. difficile on a concentration basis.

**TABLE 1:** MICs for 54 clinically isolated strains of C. difficile

Antibiotic	MIC (μg/ml)		
	MIC_50_	MIC_90_	MIC range
OPS-2071	0.125	0.5	0.016–1
Ciprofloxacin	32	128	16–128
Levofloxacin	128	>128	4 to >128
Vancomycin	2	4	1 to >128
Metronidazole	2	4	1 to >128
Fidaxomicin	0.063	8	0.016 to >128

### Antibacterial activity comparison between 20 hypervirulent and 42 nonhypervirulent strains of C. difficile.

In order to compare the antibacterial activity of OPS-2071 between hypervirulent and nonhypervirulent strains, 20 hypervirulent and 42 nonhypervirulent strains were obtained from the University of Western Australia (Perth, Australia) and Rakuno Gakuen University (Hokkaido, Japan). C. difficile can be characterized by PCR ribotyping (8). Several ribotypes are known to be epidemiologically hypervirulent, high-toxin-producing strains. In addition, these strains were reported to be highly resistant to existing fluoroquinolones (1, 18–20). We tested the *in vitro* antibacterial activity against 62 strains of C. difficile, which were genetically characterized and different from the 54 strains in Table 1, including 20 epidemiologically hypervirulent strains (PCR ribotype 018, 2 strains; 023, 2 strains; 027, 4 strains; 056, 2 strains; 078, 7 strains; and 244, 3 strains) (6, 7, 21–23). There was no significant difference in the susceptibility to OPS-2071 and vancomycin between hypervirulent and nonhypervirulent strains (Table 2); however, significant differences in susceptibility to fidaxomicin (*P < *0.01) and metronidazole (*P < *0.05) were found (Wilcoxon rank sum test). The ribotypes of these 20 hypervirulent strains are shown in Table S2.

**TABLE 2:** MIC distribution of each drug against hypervirulent and nonhypervirulent strains

MIC (μg/ml)	Cumulative percentage of 62 strains (%)^*a*^							
	OPS-2071		Vancomycin		Fidaxomicin		Metronidazole	
	Hyp (*n* = 20)	Non-hyp (*n* = 42)	Hyp (*n* = 20)	Non-hyp (*n* = 42)	Hyp (*n* = 20)	Non-hyp (*n* = 42)	Hyp (*n* = 20)	Non-hyp (*n* = 42)
<0.004						0		
0.008						5		
0.016	0	0				5		
0.031	65	79				10		
0.063	75	88			0	52		
0.125	80	90			40	86	0	0
0.25	100	100		0	90	98	45	74
0.5			0	10	100	100	85	95
1			95	81			95	100
2			100	98			95	
4				100			95	
8							95	
>16							100	

Measure	MIC values (μg/ml)							

MIC range	0.03–0.25	0.03–0.25	1–2	0.5–4	0.125–0.5	0.008–0.5	0.25–16	0.25–1
MIC_50_	0.03	0.03	1	1	0.25	0.06	0.5	0.25
MIC_90_	0.25	0.125	1	2	0.25	0.25	1	0.5

a MIC values for hypervirulent strains (hyp) and nonhypervirulent (non-hyp) strains were compared for statistical significance using a two-tailed Wilcoxon rank sum test (*P *< 0.05). *n*, number of strains which were tested.

### Bactericidal activity.

The inhibitory and bactericidal activities of OPS-2071 and other drugs against 10 of the 54 clinical isolates are summarized in Table 3. These 10 clinical isolates were selected from 44 of 54 clinical isolates, described in Table 1, based on the inclusion criterion of a smaller MIC_90_ to OPS-2071, vancomycin, metronidazole, and fidaxomicin. The minimum bactericidal concentration of OPS-2071 required to eradicate 90% of organisms (MBC_90_) was the same as the MIC_90_. In contrast, the MBC_90_ of the other drugs was 1- to 2-fold higher than the corresponding MIC_90_ values. These data indicate that OPS-2071 has bactericidal activity against clinically isolated strains of C. difficile at a concentration equivalent to the MIC.

**TABLE 3:** Comparison of MICs and MBCs of OPS-2071 and other compounds against C. difficile^*a*^

Compound	MIC		MBC		MBC_90_/MIC_90_ (μg/ml)
	Range (μg/ml)	MIC_90_	Range (μg/ml)	MBC_90_	
OPS-2071	0.03–0.5	0.5	0.03–0.5	0.5	1
Vancomycin	2–4	4	4–8	8	2
Metronidazole	1–4	4	1–4	4	1
Fidaxomicin	0.06–0.25	0.125	0.06–0.25	0.25	2

a Of the 54 clinical isolates used in Table 1, 10 were selected and used for this test.

### Inhibition of DNA gyrase activity.

DNA gyrase and DNA topoisomerase IV are, respectively, the primary and secondary targets of fluoroquinolones. In C. difficile, evidence exists that the secondary target is absent (24). To evaluate the mechanism of action, the ability of OPS-2071 to inhibit DNA gyrase was evaluated and compared with that of existing quinolones in C. difficile. As expected, the 50% inhibitory concentration (IC_50_ [μg/ml]) of OPS-2071 was much lower than those of ciprofloxacin and levofloxacin (OPS-2071 IC_50_, 0.23 [range, 0.17–0.32]; ciprofloxacin IC_50_, 8.59 [4.23–22.29]; levofloxacin IC_50_, 11.49 [6.08–46.29]).

### Killing kinetics.

**FIG 2:**
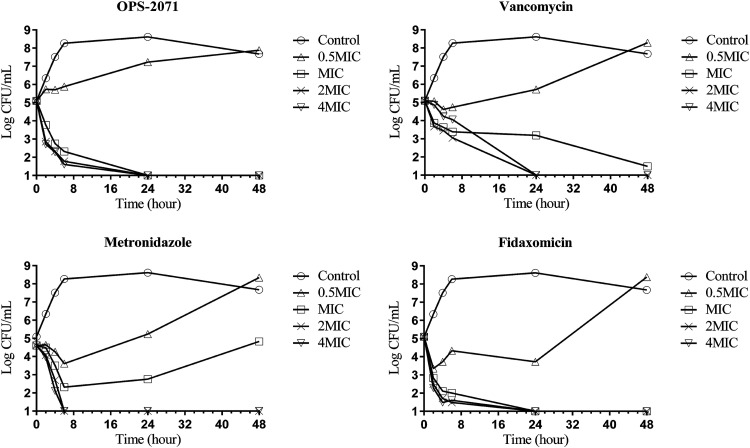
*In vitro* time-kill curve against C. difficile. Time-kill curves were determined using C. difficile ATCC 700057 in GAM broth at concentrations of 0.5-, 1-, 2-, and 4-fold MIC. The test tubes were incubated for 48 h. The CFU/ml of the bacterial suspensions were calculated at 0, 2, 4, 6, 24, and 48 h on GAM agar plates under anaerobic conditions.

Time-kill curves for OPS-2071, vancomycin, metronidazole, and fidaxomicin were determined using C. difficile ATCC 700057 (Fig. 2). In order to do this assay, the MICs of OPS-2071, vancomycin, metronidazole, and fidaxomicin against the strain were retested for this assay, and the MICs determined by a broth dilution method were 0.03, 4, 0.5, and 0.03 μg/ml, respectively. After the addition of OPS-2071 at concentrations higher than the MIC, the numbers of viable bacteria decreased rapidly, with a 2-log reduction in the first 2 h and a continued decrease to an undetectable level within 24 h. After 48 h of incubation, regrowth of the bacteria did not occur. The killing kinetics of fidaxomicin were close to those of OPS-2071. As for vancomycin and metronidazole, these drugs needed over 2-fold MIC for an undetectable level to be reached. The killing kinetics of OPS-2071 showed that the drug is effective against C. difficile.

### Frequency of spontaneous resistance.

To test the frequency of spontaneous resistance, the MICs were determined for this assay. The MICs of OPS-2071, vancomycin, and fidaxomicin against C. difficile ATCC 700057 were 0.03, 2, and 0.06 μg/ml, respectively. The frequency of spontaneous resistance to OPS-2071 and other compounds are shown in Table 4. No mutants were observed at concentrations of 4-, 16-, and 64-fold MIC of OPS-2071 with a frequency of less than 9.17 × 10^−9^. In contrast, resistant mutants were observed at 4-fold MIC of fidaxomicin. C. difficile strain ATCC 700057 did not show a propensity to develop spontaneous resistance in response to OPS-2071.

**TABLE 4:** Frequency of spontaneous resistance against C. difficile ATCC 700057

Compound	Dose	Test concn (μg/ml)	Frequency
OPS-2071	4× MIC	0.125	<9.17 × 10^–9^
	16× MIC	0.5	<9.17 × 10^–9^
	64× MIC	2	<9.17 × 10^–9^
Vancomycin	4× MIC	8	<9.17 × 10^–9^
	16× MIC	32	<9.17 × 10^–9^
	64× MIC	128	<9.17 × 10^–9^
Fidaxomicin	4× MIC	0.25	1.47 × 10^–7^
	16× MIC	1	<9.17 × 10^–9^
	64× MIC	4	<9.17 × 10^–9^

### Mutant prevention concentration.

The mutant prevention concentration (MPC) against C. difficile ATCC 700057 is defined as the minimal concentration at which there is no emergence of resistant bacteria (25). The MPC of OPS-2071, vancomycin, and fidaxomicin are 0.5, 8, and 8 μg/ml, respectively. The MPC of OPS-2071 was the lowest among tested compounds. This study suggests that the risk of emergence of OPS-2071-resistant C. difficile strains is considerably lower than that for vancomycin and fidaxomicin.

### Postantibiotic effect of OPS-2071.

The postantibiotic effect (PAE) is defined as the delayed regrowth or the persistent growth suppression of bacteria after short antimicrobial exposure. The PAEs of OPS-2071 against C. difficile were determined and compared with those of other drugs (Table 5). A broth method was used to determine the MICs of OPS-2071, vancomycin, and fidaxomicin against C. difficile ATCC 700057. The MICs were 0.06, 4, and 0.06 μg/ml, respectively, and the PAEs of OPS-2071 at 4- and 8-fold the MIC against C. difficile (3.93 to 4.04 h) were longer than those of vancomycin (1.03 to 1.25 h) and fidaxomicin (2.86 to 2.77 h). As OPS-2071 showed a longer PAE against C. difficile than either vancomycin or fidaxomicin, the duration of OPS-2071’s antibacterial efficacy against C. difficile can be expected to be longer than that of the other two agents.

**TABLE 5:** Postantibiotic effect of OPS-2071 against C. difficile ATCC 700057 (hours)

Dose	OPS-2071	Vancomycin	Fidaxomicin
4× MIC (μg/ml)	3.93	1.03	2.86
8× MIC (μg/ml)	4.04	1.25	2.77

### Cytotoxicity assay.

The cytotoxicity of OPS-2071 was determined with the neutral red uptake assay in BALB/3T3 clone A31 cells. OPS-2071 did not show cytotoxicity up to 100 μg/ml (IC_50_, >100 μg/ml).

### Pharmacokinetics in the hamster.

**FIG 3:**
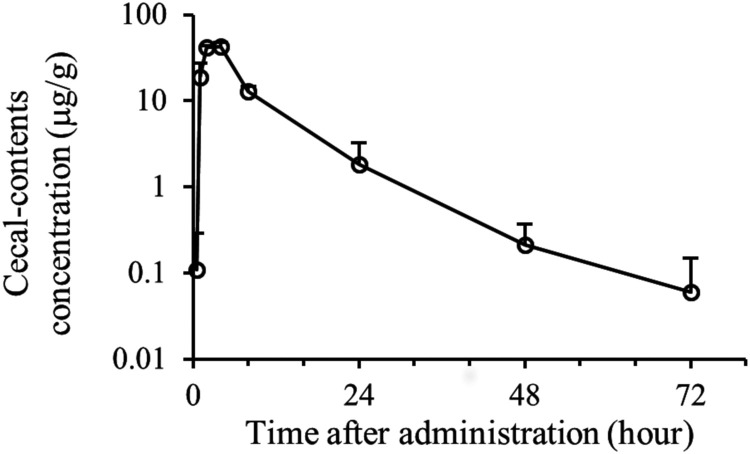
Time-concentration profile of OPS-2071 in cecal contents after oral administration of OPS-2071 to hamsters. OPS-2071 was orally administered at 2 mg/kg to male Syrian hamsters. The concentration in cecal contents was determined 0.5, 1, 2, 4, 8, 24, 48, and 72 h postdose (*n* = 5 for each time point, mean + standard deviation). The Cmax was 42.95 μg/g at 4 h postdose.

OPS-2071 was orally administered at 2 mg/kg. The maximum concentration (Cmax) in the cecal contents reached 42.95 μg/g at 4 h postdose and then decreased to 1.84 μg/g at 24 h postdose (Fig. 3). This concentration was many times higher than the MIC_90_ and MBC_90_ of OPS-2071 for C. difficile.

### Pharmacokinetics in the rat.

**FIG 4:**
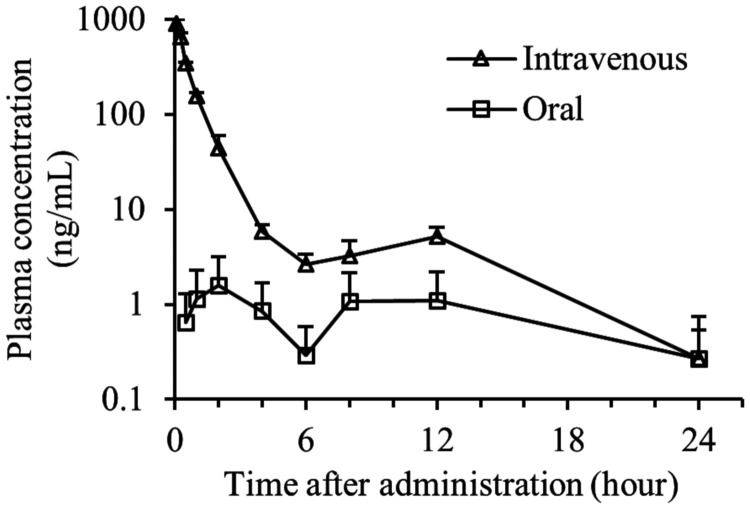
Time-concentration profile of OPS-2071 in plasma after oral and intravenous administration to rats. OPS-2071 was orally or intravenously administered at 1 mg/kg to male SD rats, and then plasma was collected. The concentration of OPS-2071 in plasma was determined by a validated LC-ESI-MS/MS method (mean + standard deviation, *n* = 3), and then bioavailability was calculated. The concentrations at 48 and 72 h after administration were <0.5 ng/ml. The curve showed a bimodal plasma concentration time profile due to bile excretion.

**FIG 5:**
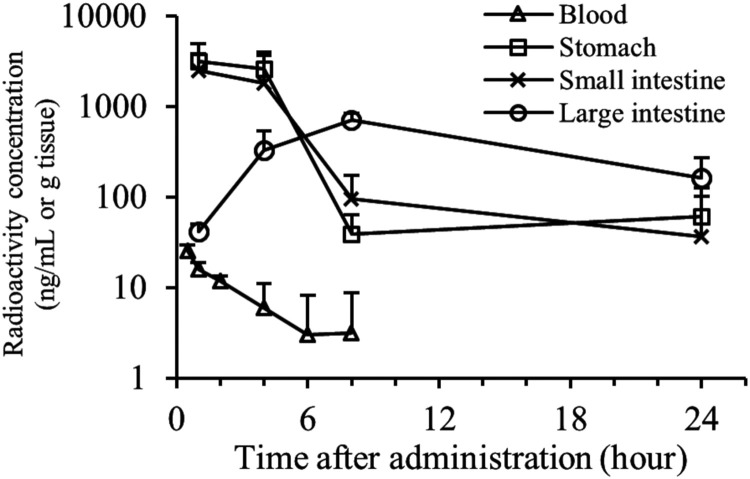
Time-concentration profile of radioactivity in plasma and gastrointestinal tract after oral administration of 14C-OPS-2071 to rats. ^14^C-OPS-2071 was orally administered at 3 mg/kg to male SD rats, and the blood, stomach, small intestine, and large intestine were collected. Radioactivity was measured using a liquid scintillation counter to calculate the concentration (mean + standard deviation, *n* = 3). No radioactivity was detected at 24 h postdose (<3× background radioactivity) for all matrices.

OPS-2071 showed low systemic exposure, with values for the area under the concentration-time curve calculated to the last observable concentration at time *t* (AUC_t_) after oral and intravenous administration of OPS-2071 at 1 mg/kg of 19.48 and 671.7 ng · h/ml, respectively (Fig. 4). Therefore, the calculated oral bioavailability was 2.9%. In addition, orally administered ^14^C-OPS-2071 derived radioactivity was distributed to the large intestine at a high concentration (Fig. 5), and more than 95% of dosed radioactivity was excreted in feces. These results indicate that the low absorption of OPS-2071 makes it suitable for treating CDI.

### *In vivo* activity in the hamster.

**FIG 6:**
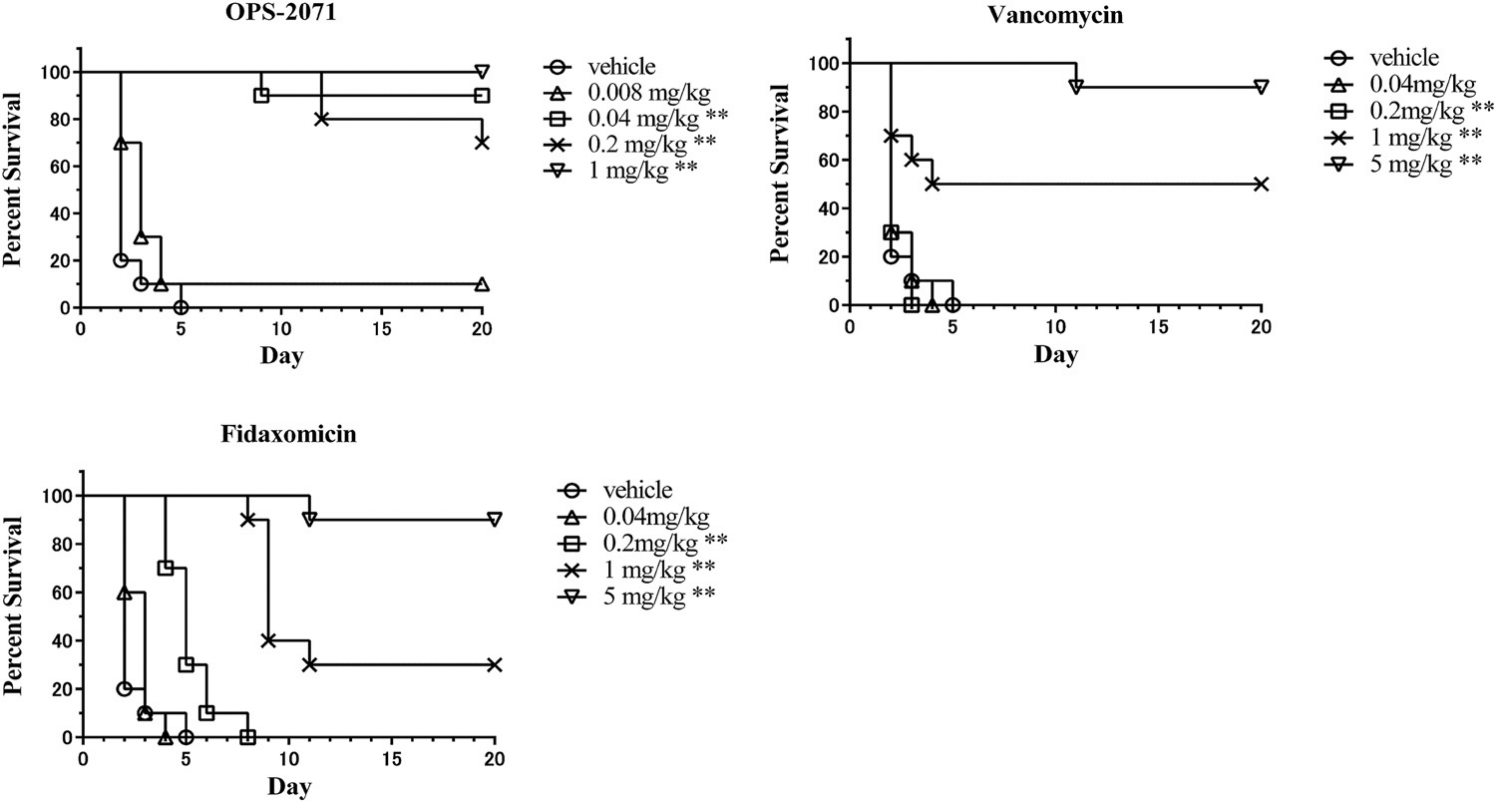
Efficacy of OPS-2071 and other drugs in a hamster model of CDI. Survival curve of hamsters after C. difficile ATCC 43255 infection followed by administration of clindamycin and subsequent treatment. Ten mice were used in each group. Results of a log-rank test with Dunnett-type comparison versus vehicle; *, *P* < 0.05; **, *P* < 0.01. The ED_50_ on day 20 were calculated using the probit method.

The effectiveness of OPS-2071 and other drugs was assessed in a hamster model of CDI. This model is the current gold standard used to assess potential efficacy in the treatment of CDI (26–29). The survival curve for each drug tested in this study is shown in Fig. 6. After infection, all hamsters treated with vehicle died within 5 days. Compared with the vehicle control, significant efficacy was observed for OPS-2071 at dosages of 0.04 mg/kg and above. In contrast, significant efficacy was observed for vancomycin and fidaxomicin only at dosages of 1 mg/kg and above. The 50% effective doses (ED_50_) of OPS-2071, vancomycin, and fidaxomicin were 0.0313 mg/kg/day (95% confidence interval [CI], 0.0131 to 0.0686 mg/kg/day), 1.22 mg/kg/day (95% CI, 0.597 to 2.39 mg/kg/day), and 1.63 mg/kg/day (95% CI, 0.836 to 3.20 mg/kg/day), respectively. OPS-2071 also had an ED_50_ 39.0-fold lower than that of vancomycin and 52.1-fold lower than that of fidaxomicin.

## DISCUSSION

C. difficile has been reported as an urgent threat to human health by the Centers for Disease Control and Prevention in the United States since 2013 (30). To date, new compounds and new therapies have been developed to treat CDI (16, 26, 31). However, there is no drug presently able to overcome such problems as fulminant life-threatening colitis characterized by high mortality (35 to 50%) and a high rate of recurrence, for which CDI is the cause in 15 to 25% of initial episodes (4). FMT is a new approach showing high cure rates for recurrent CDI (32); however, wide clinical use of FMT is still difficult because the preferred method for FMT administration is yet to be defined (32). Therefore, it is necessary to develop an easy-to-use CDI treatment such as antibiotics used for common infectious diseases. Fluoroquinolone has been used in clinical practice for over 30 years and is well known to be an antibiotic with high killing efficiency against both Gram-positive and Gram-negative bacteria. Regarding adverse events, it is generally well tolerated, and most adverse effects are mild (33). Though fluoroquinolone is still considered to be an important antibiotic for patients with serious infections, the FDA issued changes to the safety labeling for systemic fluoroquinolone in 2018. Apart from the existing major well-known central nervous system (CNS)-related adverse effects (phototoxicity, QT interval prolongation, and diarrhea), the FDA required that the risk of serious blood sugar disturbances and psychiatric side effects be added to the labeling (34). It is important to note that most of these adverse events, including the newly added risks, are caused by the systemic absorption of antibiotics. Fluoroquinolones are considered to be one of the major antibiotic classes that can induce CDI because predominant strains are resistant to common fluoroquinolones such as ciprofloxacin and levofloxacin (1, 18–20). We approached the development of a new antibiotic with the aim of increasing antibacterial activity against C. difficile by utilizing the quinolone structure and decreasing the oral absorption in order to reduce the risk of adverse events associated with fluoroquinolone class antibiotics. Based on our experience in the area of structure-activity relationships, we succeeded in developing OPS-2071 as a novel fluoroquinolone that demonstrates potent antibacterial activity against C. difficile.

The *in vitro* activity against clinical isolates was similar to that of fidaxomicin and more potent than that of vancomycin. The MIC_90_ of fidaxomicin for 54 clinical isolates was 8 μg/ml (Table 1), and the MIC_90_ for 62 strains was 0.25 μg/ml (Table 2). In contrast to these 62 strains, the 54 clinical isolates included fidaxomicin-resistant strains, and this resulted in the different fidaxomicin MIC_90_ values. This indicates that there is a greater range of MICs for fidaxomicin in clinical isolates compared to OPS-2071. Furthermore, while there is no difference in the activity of OPS-2071 between hypervirulent and nonhypervirulent strains, a significant difference in susceptibility to fidaxomicin was observed. Also, MICs of OPS-2071 are not influenced by pH (Table S3). The intraluminal pH changes rapidly from highly acid in the stomach to about pH 6.0 in the duodenum, and the pH gradually increases in the small intestine from pH 6.0 to about pH 7.4 in the terminal ileum. The activity of OPS-2071 is not affected by differences in pH in the gastrointestinal tract.

In addition, OPS-2071 demonstrated bactericidal activity against C. difficile at concentrations close to MIC, rapidly reducing the number of the organisms with no regrowth observed after 48 h (Fig. 2). Although each time point represents a single sample, these killing kinetics are reasonable because the trajectories of the kinetics depend on the concentration (MIC values) of these compounds. Since OPS-2071 showed the longest PAE among the tested compounds, this and the other characteristics of OPS-2071 contributed to the ideal bactericidal activity curve portrayed by its inhibition of bacterial regrowth *in vitro*.

With regard to resistance, the frequency of spontaneous resistance was extremely low for OPS-2071, despite inoculation with very high concentrations of C. difficile. No spontaneous resistant mutants grew at 4-, 16- or 64-fold the MIC. Furthermore, the MPC value, which has been employed in the evaluation of an antibiotic’s ability to minimize or limit the development of resistant organisms, was the lowest among all tested compounds. The data suggest that C. difficile may not readily develop resistance to OPS-2071 in the clinical setting and that the drug is suitable as a monotherapy for the treatment of CDI.

Regarding the pharmacokinetics of OPS-2071, the bioavailability at 1 mg/kg in rats was 2.9%, indicating that OPS-2071 has a low absorption profile in rats. We speculated that this profile may also apply to hamsters, because a high concentration of OPS-2071 was observed in the cecal contents of hamsters (Fig. 3). This characteristic of low absorption of OPS-2071 is desirable for increasing therapeutic efficacy against the onset of C. difficile while at the same time reducing the risk of systemic adverse events, reinforcing the evidence that OPS-2071 should be an effective agent for treating CDI.

With respect to the efficacy in animal models, many therapeutic agents have been tested in the hamster model of clindamycin-induced CDI (26–29). Specifically, we organized our animal model with reference to an animal model in which clindamycin is administered 1 day after the C. difficile infection (27), because CDI is normally induced by administration of antibiotics with preexisting C. difficile in the intestine. In addition, the MIC of C. difficile ATCC 43255 to clindamycin is reported as 8 μg/ml (35). Since test compounds were administered for 5 days after 1 mg/kg clindamycin administration, the effect of the clindamycin on other test compounds appears to be very low.

OPS-2071 showed significant efficacy in this model with an effective dose, which was 39.0-fold and 52.1-fold lower than those of vancomycin and fidaxomicin, respectively. Although the *in vitro* antibacterial activity of OPS-2071 against C. difficile was similar to that of fidaxomicin, *in vivo* efficacy was demonstrated at a lower dose than that of fidaxomicin. Similar to OPS-2071, absorption of vancomycin and fidaxomicin is also low, and both reach the colon efficiently. The oral bioavailability of vancomycin is <10%, and fidaxomicin is minimally absorbed, with plasma concentrations in the low nanogram per milliliter range or lower (36, 37). The pharmacokinetic (PK) profiles of these three drugs are fairly similar. These results suggested the possibility that the cecal contents affect the antibacterial activity among these three drugs in different manners.

For the data lacking biological replicates in this paper, the limitation as a single data set must be considered. However, we believe that the data are sufficiently reliable considering that they have dose response, time course-dependent changes, or large data sets.

In conclusion, OPS-2071 demonstrated potent *in vitro* activity against clinical isolates and hypervirulent strains. *In vivo* efficacy using hamsters was shown at doses 39.0- and 52.1-fold lower than those of vancomycin and fidaxomicin. The data support that OPS-2071 has potential as an agent for treating CDI. The current challenge in treating CDI is the frequency of recurrence (38). One of the main causes of recurrence is the dormancy of C. difficile as spores (39), which can revert back to being an active infection. It is unknown if OPS-2071 eliminates C. difficile, including its spores, an important property central to reducing infection recurrence. Follow-up studies are needed to explore this matter in more depth.

## MATERIALS AND METHODS

### Antibiotics.

OPS-2071 was synthesized by Otsuka Pharmaceuticals. Co., Ltd. (Tokushima, Japan). ^14^C-OPS-2071 was synthesized by Curachem, Inc. (Chungcheongbuk-do, South Korea). Ciprofloxacin and levofloxacin were purchased from Sigma-Aldrich (St. Louis, MO). Vancomycin was purchased from Fujifilm Wako Pure Chemical Corp. (Osaka, Japan). Fidaxomicin was purchased from Optimer Pharmaceuticals, Inc. (Jersey City, NJ) and extracted and purified at Otsuka Pharmaceuticals. Co., Ltd.

### Microorganisms.

Clinically isolated strains were obtained with 112 strains in total. We obtained 4 strains from Aino Hospital (Osaka, Japan) and 50 strains from Miroku Medical Laboratory (Nagano, Japan) as shown in Table 1. A total of 17 hypervirulent and 18 nonhypervirulent strains were obtained from the University of Western Australia (Perth, Australia), and 3 hypervirulent and 24 nonhypervirulent strains were obtained from Rakuno Gakuen University (Hokkaido, Japan) as shown in Table 2. ATCC 700057 and ATCC 43255 were purchased from the American Type Culture Collection (ATCC; Manassas, VA).

### Animals.

All studies were carried out in adherence to the Guidelines for Animal Care and Use (40), which were approved by the Animal Care and Use Committee of Otsuka Pharmaceutical Co., Ltd.

Four to five-week-old, specific-pathogen-free, male golden Syrian hamsters were purchased from Japan SLC, Inc. (Shizuoka, Japan). Five- to six-week-old, specific-pathogen-free, male Sprague-Dawley (SD) rats were purchased from Charles River Laboratories Japan, Inc. Hamsters were housed in mouse Hi-PSF cages (Clea Japan, Inc.) during the infection study and Econ TPX cages (Clea Japan, Inc.) during the PK study. SD rats were housed in stainless bracket cages (Nihon Cage Co, Ltd.) and Econ TPX cages (Clea Japan, Inc.) Animals were fed with a certified diet (CRF-1; Oriental Yeast Co., Ltd.) *ad libitum*. Water, food, and bedding were autoclaved prior to use. Animals were allowed to acclimate in the animal facility, in which environmental controls were set to the following conditions: a temperature of 23 ± 2°C, humidity of 60% ± 10%, and a 12 h light-dark cycle (light period, 7 a.m. to 7 p.m.) for more than 1 week. Ten randomly selected hamsters per group were used for the experimental infection model. Five hamsters per group and three rats per group, randomly selected, were used for the pharmacokinetic study.

### Determination of antibacterial activity.

MICs obtained using the agar dilution method were determined visually as described for the CLSI method (41, 42). MICs obtained using the broth dilution method were determined visually based on the CLSI method except for using Gifu anaerobic medium (GAM) broth instead of supplemented Brucella broth (41, 42).

### MBC testing.

From among 54 strains, the 10 C. difficile strains sensitive to the antibiotics being tested were selected for the MBC test. Using the agar dilution method, these sensitivities were determined based on the MIC for OPS-2071, vancomycin, fidaxomicin, and metronidazole as shown in Table 1. In this test, MIC testing was performed using the broth dilution method as recommended in the CLSI (41). After the MICs were determined, 10 μl of the bacterial suspension from the MIC test tube was inoculated onto agar plates and incubated at 37°C. The bacterial colonies were then counted and bacterial numbers were calculated. The MBC was considered to be the lowest concentration of the antibiotics that prevented growth and reduced the inoculum by >99.9% within 48 h, irrespective of counts of survivors at higher antibiotic concentrations.

### Killing kinetics.

The bacterial suspensions of C. difficile ATCC 700057 in GAM broth were precultured for 2 h, and the test agents were added to the test tubes at concentrations of 0.5-, 1-, 2-, and 4-fold MIC. The test tubes were incubated for 48 h. The CFU/ml values of the bacterial suspensions were calculated at 0, 2, 4, 6, 24, and 48 h by culturing 0.1-ml samples of serial 10-fold dilutions on GAM agar plates at 37°C under anaerobic conditions and then counting the number of growing colonies. The CFU/ml value at each time point is from one sample of datum.

### Inhibition of DNA gyrase activity.

The subunits A and B of DNA gyrase of C. difficile were purchased from Inspiralis Ltd. (Norwich, UK). The supercoiling activity of DNA gyrase was determined as described previously (43). The inhibitory effect of compounds was assessed by determining the concentration required to inhibit 50% of the enzyme (IC_50_).

The gyrase supercoiling reaction mixtures (30 μl), which contained gyrase (1 unit) and relaxed pBR322 DNA (500 ng), with or without drug solution, were incubated at 37°C for 30 min. The assay reaction mixture was analyzed by electrophoresis. The gels were stained with ethidium bromide, and the density of the supercoiled plasmid was obtained using a UV illuminator. The image of the supercoiled plasmid was analyzed using image processing software (ImageJ 1.50i; National Institutes of Health, USA). The IC_50_ was defined as the concentration that caused 50% inhibition of the supercoiled plasmid.

### Frequency of spontaneous resistance.

The frequency of spontaneous C. difficile ATCC 700057 resistance to OPS-2071, vancomycin, metronidazole, and fidaxomicin was measured by inoculating the bacterial suspension onto a supplemented Brucella agar (Brucella agar with 5 μg/ml of hemin, 1 μg/ml of vitamin K1, and 5% laked horse blood) plate containing antibiotics at 4-, 16-, and 64-fold the agar dilution MIC. The frequency of spontaneous resistance to each compound was calculated as the number of resistant colonies formed per the number of inoculated bacteria.

### Mutant prevention concentration.

The mutant prevention concentration was measured by plating approximately 10^10^ CFU of C. difficile ATCC 700057 onto a GAM agar plate containing the test agent. Inoculated plates were incubated for 3 days, and the MIC for each antibiotic that prevented the growth of colonies was determined using the agar dilution method as described by the CLSI (41). The MIC values of grown colonies were compared to determine which bacteria were resistant. The MPC was defined as the lowest concentration that prevented the growth of resistant bacteria.

### Determination of PAE.

C. difficile ATCC 700057 was cultured in GAM agar and adjusted in GAM broth to approximately 10^6^ CFU/ml. The bacterial suspensions were preincubated at 37°C for 1 h. Before and after preincubation, the CFU/ml was determined using serial cultures of 10-fold dilutions on GAM agar at 37°C. After preincubation, test and reference solutions were added to each bacterial suspension in a test tube, and the suspensions were cultured for 1 h. After exposure to the test compounds, bacterial suspensions were washed with GAM broth and cultured for 6 h. In order to calculate the CFU/ml of these suspensions every hour, serial 10-fold dilutions were cultured on agar plates. The PAE was defined according to Craig and Gudmundsson as PAE = *T* − C, where *T* is the time required for the viable counts of the exposed bacteria to increase by 1 log_10_ above the counts observed immediately after washing, and *C* is the corresponding time for the antibiotic unexposed controls (44). The PAEs of OPS-2071 were compared with those of other compounds at 4× and 8× the MIC.

### Cytotoxicity assay.

The BALB/c mouse fibroblast cell line, BALB/3T3 clone A31 cell (JCRB9005), was obtained from the Health Science Research Resources Bank (Osaka, Japan) and was cultured in Dulbecco’s modified Eagle’s medium (Sigma-Aldrich) supplemented with 10% heat-inactivated newborn calf serum (Gibco), 100 IU/ml penicillin and 100 μg/ml streptomycin (Sigma-Aldrich). The neutral red uptake assay was adapted to determine cytotoxicity as follows (45). The cells were seeded into a 96-well plate at 1 × 10^4^ cells/well in 100 μl of culture medium and incubated for 25 h at 37°C in 5% CO_2_. After the culture medium was removed, cells were treated with negative control (dimethyl sulfoxide) and OPS-2071 (1.77 to 100 μg/ml, six wells/dose) in Earl’s balanced salt solution (EBSS; Sigma-Aldrich) supplemented with 10 mmol/liter *N*-(2-hydroxyethyl)piperazine-*N*′-(2-ethanesulfonic acid) (HEPES; Sigma-Aldrich) for 1 h and 50 min, followed by incubation with culture medium for 21 h. The culture medium was replaced with 50 μg/ml neutral red (Sigma-Aldrich) containing culture medium and incubated for 3 h. The cells were washed with Dulbecco’s phosphate-buffered saline (Gibco), and neutral red was extracted with extraction solution (50% ethanol, 49% water, and 1% acetic acid). Absorbance at 540 nm was measured using a Molecular Devices Emax plate reader (Molecular Devices, San Jose, CA), and cell viability was calculated.

### Pharmacokinetics in the hamster.

OPS-2071 was orally administered at 2 mg/kg to male Syrian hamsters, and the cecal contents were collected 0.5, 1, 2, 4, 8, 24, 48, and 72 h postdose (*n* = 5 for each time point). After homogenizing with saline and treatment using acetonitrile-formic acid (100:1 [vol/vol]), the OPS-2071 concentration in cecal contents was determined using a validated liquid chromatography-electrospray ionization-tandem mass spectrometry (LC-ESI-MS/MS) method. The mass spectrometer 4000 QTRAP (AB Sciex Pte. Ltd.) and high-performance liquid chromatography Prominence UFLC system (Shimadzu Corp.) were used. The pharmacokinetic (PK) parameters (Cmax) were determined with WinNonlin professional software version 6.3, (Pharsight Corp.).

### Pharmacokinetics in the rat.

OPS-2071 was orally or intravenously administered at 1 mg/kg to male SD rats, and plasma was collected 0.5, 1, 2, 4, 6, 8, 12, 24, 48, and 72 h postdose (oral, *n* = 3 for each time point) or 0.083, 0.25, 0.5, 1, 2, 4, 6, 8, 12, 24, 48, and 72 h postdose (intravenous, *n* = 3 for each time point). After solid-phase extraction, the OPS-2071 concentration in plasma was determined by a validated LC-ESI-MS/MS method, and bioavailability was calculated. The mass spectrometer QTRAP 5500 (AB Sciex Pte. Ltd.) and high-performance liquid chromatography Prominence UFLCXR system (Shimadzu Corp.) were used. The PK parameters (AUC_t_) were determined with the same WinNonlin professional software, and the bioavailability was calculated from AUC_t,oral_ and AUC_t,intravenous_.

^14^C-OPS-2071 was orally administered at 3 mg/kg to male SD rats. Blood was collected 1, 2, 4, 6, 8, 12, 24, 72, and 168 h postdose (*n* = 3 for each time point), the stomach, small intestine, and large intestine were collected 1, 4, 8, 24, 72, and 168 h postdose (*n* = 3 for each time point), and feces were collected up until 168 h postdose (*n* = 3). The collected blood, stomach, small intestine, large intestine, and feces were dissolved in a tissue solubilizer, and radioactivity was measured using a liquid scintillation counter LSC-6101 (Aloka Co., Ltd.) to calculate the concentration. Cumulative excretion in feces was calculated from fecal radioactivity concentrations.

### *In vivo* hamster model of C. difficile infection.

C. difficile ATCC 43255 was cultured on GAM agar, and a bacterial suspension was prepared in saline (26). The MIC values of OPS-2071, vancomycin, and fidaxomicin against C. difficile ATCC 43255 are 0.016, 2, and 0.06, respectively. Hamsters were infected using oral gavage with 0.5 ml of suspension containing approximately 10^6^ CFU of C. difficile (day −1). The next day, the animals were administered clindamycin phosphate (0.2 mg/ml in water) by oral gavage at a dose of 5 ml/kg of body weight, for a final dose of 1 mg/kg (day 0). The animals were allocated to the test groups using a stratified randomization method based on the body weight of each infected animal. The test compounds were administered by oral gavage once daily for 5 days (OPS-2071, 0.008 to 1 mg/kg; fidaxomicin, 0.04 mg/kg to 5 mg/kg; vancomycin, 0.04 to 5 mg/kg [5-fold dilution]). Infected control animals were administered 5% (wt/vol) gum arabic solution by oral gavage. Animals were observed at least once daily for mortality and the presence or absence of diarrhea, and mortality was recorded once daily. From the perspective of animal protection, any significantly debilitated hamsters were euthanized for humane reasons. The cecal contents of dead animals were tested for toxin A and toxin B using C.Diff Quik Chek Complete (Abbott Diagnostics Medical Co., Ltd., Tokyo, Japan) to confirm that the death of the animal was due to CDI.

### Statistical analysis.

Statistical analyses were conducted using SAS software release 9.3 (SAS Institute Japan). The significance level of the test was set at 5%.

Differences between hypervirulent and nonhypervirulent strains were statistically analyzed using two-tailed Wilcoxon rank sum tests.

The statistical significance of OPS-2071, fidaxomicin, and vancomycin treatment was analyzed at all doses against vehicle. Survival curves were estimated for each group using the Kaplan-Meier method. Differences in survival homogeneity in each group were evaluated with a log-rank test with Dunnett-type (two-sided) comparison.

The effective dose at 50% (ED_50_) of the dose response was generated for OPS-2071, vancomycin, and fidaxomicin on day 20, and its 95% confidence interval was determined using a probit method.

## Supplementary Material

Supplemental file 1

## References

[1] Leffler DA, Lamont JT. 2015. Clostridium difficile infection. N Engl J Med 373:287–288. doi:10.1056/NEJMc1506004. 26176396

[2] Asha NJ, Tompkins D, Wilcox MH. 2006. Comparative analysis of prevalence, risk factors, and molecular epidemiology of antibiotic-associated diarrhea due to Clostridium difficile, Clostridium perfringens, and Staphylococcus aureus. J Clin Microbiol 44:2785–2791. doi:10.1128/JCM.00165-06. 16891493 PMC1594656

[3] Lessa FC, Mu Y, Bamberg WM, Beldavs ZG, Dumyati GK, Dunn JR, Farley MM, Holzbauer SM, Meek JI, Phipps EC, Wilson LE, Winston LG, Cohen JA, Limbago BM, Fridkin SK, Gerding DN, McDonald LC. 2015. Burden of Clostridium difficile infection in the United States. N Engl J Med 372:825–834. doi:10.1056/NEJMoa1408913. 25714160 PMC10966662

[4] Guery B, Galperine T, Barbut F. 2019. Clostridioides difficile: diagnosis and treatments. BMJ 366:l4609. doi:10.1136/bmj.l4609. 31431428

[5] Fischer S, Uckert AK, Landenberger M, Papatheodorou P, Hoffmann-Richter C, Mittler AK, Ziener U, Hagele M, Schwan C, Muller M, Kleger A, Benz R, Popoff MR, Aktories K, Barth H. 2020. Human peptide alpha-defensin-1 interferes with Clostridioides difficile toxins TcdA, TcdB, and CDT. FASEB J 34:6244–6261. doi:10.1096/fj.201902816R. 32190927

[6] Bauer MP, Notermans DW, van Benthem BHB, Brazier JS, Wilcox MH, Rupnik M, Monnet DL, van Dissel JT, Kuijper EJ. 2011. Clostridium difficile infection in Europe: a hospital-based survey. Lancet 377:63–73. doi:10.1016/S0140-6736(10)61266-4. 21084111

[7] Walker AS, Eyre D, Wyllie D, Dingle K, Griffiths D, Shine B, Oakley S, O'Connor L, Finney J, Vaughan A, Crook D, Wilcox M, Peto TA, Infections in Oxfordshire Research Database. 2013. Relationship between bacterial strain type, host biomarkers, and mortality in Clostridium difficile infection. Clin Infect Dis 56:1589–1600. doi:10.1093/cid/cit127. 23463640 PMC3641870

[8] Fatima R, Aziz M. 2019. The hypervirulent strain of Clostridium difficile: NAP1/B1/027: a brief overview. Cureus 11:e3977. doi:10.7759/cureus.3977. 30967977 PMC6440555

[9] Bilverstone TW, Minton NP, Kuehne SA. 2019. Phosphorylation and functionality of CdtR in Clostridium difficile. Anaerobe 58:103–109. doi:10.1016/j.anaerobe.2019.102074. 31323291 PMC6699598

[10] Rodemann JF, Dubberke ER, Reske KA, Seo DH, Stone CD. 2007. Incidence of Clostridium difficile infection in inflammatory bowel disease. Clin Gastroenterol Hepatol 5:339–344. doi:10.1016/j.cgh.2006.12.027. 17368233

[11] Saidel-Odes L, Borer A, Odes S. 2011. Clostridium difficile infection in patients with inflammatory bowel disease. Ann Gastroenterol 24:263–270. 24713726 PMC3959320

[12] Tariq R, Law CCY, Khanna S, Murthy S, McCurdy JD. 2019. The impact of Clostridium difficile infection on mortality in patients with inflammatory bowel disease: a systematic review and meta-analysis. J Clin Gastroenterol 53:127–133. doi:10.1097/MCG.0000000000000968. 29206751

[13] D’Aoust J, Battat R, Bessissow T. 2017. Management of inflammatory bowel disease with Clostridium difficile infection. World J Gastroenterol 23:4986–5003. doi:10.3748/wjg.v23.i27.4986. 28785153 PMC5526769

[14] Issa M, Vijayapal A, Graham MB, Beaulieu DB, Otterson MF, Lundeen S, Skaros S, Weber LR, Komorowski RA, Knox JF, Emmons J, Bajaj JS, Binion DG. 2007. Impact of Clostridium difficile on inflammatory bowel disease. Clin Gastroenterol Hepatol 5:345–351. doi:10.1016/j.cgh.2006.12.028. 17368234

[15] McDonald LC, Gerding DN, Johnson S, Bakken JS, Carroll KC, Coffin SE, Dubberke ER, Garey KW, Gould CV, Kelly C, Loo V, Shaklee Sammons J, Sandora TJ, Wilcox MH. 2018. Clinical practice guidelines for Clostridium difficile infection in adults and children: 2017 update by the Infectious Diseases Society of America (IDSA) and Society for Healthcare Epidemiology of America (SHEA). Clin Infect Dis 66:987–994. doi:10.1093/cid/ciy149. 29562266

[16] Gupta A, Saha S, Khanna S. 2020. Therapies to modulate gut microbiota: past, present and future. World J Gastroenterol 26:777–788. doi:10.3748/wjg.v26.i8.777. 32148376 PMC7052537

[17] Clinical and Laboratory Standards Institute. 2012. Performance standards for antimicrobial susceptibility testing; twenty-second informational supplement, CLSI document M100-S22. Clinical and Laboratory Standards Institute, Wayne, PA.

[18] Pepin J, Saheb N, Coulombe MA, Alary ME, Corriveau MP, Authier S, Leblanc M, Rivard G, Bettez M, Primeau V, Nguyen M, Jacob CE, Lanthier L. 2005. Emergence of fluoroquinolones as the predominant risk factor for Clostridium difficile-associated diarrhea: a cohort study during an epidemic in Quebec. Clin Infect Dis 41:1254–1260. doi:10.1086/496986. 16206099

[19] McDonald LC, Killgore GE, Thompson A, Owens RC, Jr, Kazakova SV, Sambol SP, Johnson S, Gerding DN. 2005. An epidemic, toxin gene-variant strain of Clostridium difficile. N Engl J Med 353:2433–2441. doi:10.1056/NEJMoa051590. 16322603

[20] Loo VG, Poirier L, Miller MA, Oughton M, Libman MD, Michaud S, Bourgault AM, Nguyen T, Frenette C, Kelly M, Vibien A, Brassard P, Fenn S, Dewar K, Hudson TJ, Horn R, Rene P, Monczak Y, Dascal A. 2005. A predominantly clonal multi-institutional outbreak of Clostridium difficile-associated diarrhea with high morbidity and mortality. N Engl J Med 353:2442–2449. doi:10.1056/NEJMoa051639. 16322602

[21] DeAlmeida MN, Heffernan H, Dervan A, Bakker S, Freeman JT, Bhally H, Taylor SL, Riley TV, Roberts SA. 2013. Severe Clostridium difficile infection in New Zealand associated with an emerging strain, PCR-ribotype 244. N Z Med J 126:9–14. 24126745

[22] Lim SK, Stuart RL, Mackin KE, Carter GP, Kotsanas D, Francis MJ, Easton M, Dimovski K, Elliott B, Riley TV, Hogg G, Paul E, Korman TM, Seemann T, Stinear TP, Lyras D, Jenkin GA. 2014. Emergence of a ribotype 244 strain of Clostridium difficile associated with severe disease and related to the epidemic ribotype 027 strain. Clin Infect Dis 58:1723–1730. doi:10.1093/cid/ciu203. 24704722

[23] Miller M, Gravel D, Mulvey M, Taylor G, Boyd D, Simor A, Gardam M, McGeer A, Hutchinson J, Moore D, Kelly S. 2010. Health care-associated Clostridium difficile infection in Canada: patient age and infecting strain type are highly predictive of severe outcome and mortality. Clin Infect Dis 50:194–201. doi:10.1086/649213. 20025526

[24] Dridi L, Tankovic J, Burghoffer B, Barbut F, Petit JC. 2002. gyrA and gyrB mutations are implicated in cross-resistance to ciprofloxacin and moxifloxacin in Clostridium difficile. Antimicrob Agents Chemother 46:3418–3421. doi:10.1128/aac.46.11.3418-3421.2002. 12384345 PMC128732

[25] Lopez Y, Tato M, Gargallo-Viola D, Canton R, Vila J, Zsolt I. 2019. Mutant prevention concentration of ozenoxacin for quinolone-susceptible or -resistant Staphylococcus aureus and Staphylococcus epidermidis. PLoS One 14:e0223326. doi:10.1371/journal.pone.0223326. 31596898 PMC6785070

[26] Mathur T, Barman TK, Kumar M, Singh D, Kumar R, Khera MK, Yamada M, Inoue SI, Upadhyay DJ, Masuda N. 2018. In vitro and in vivo activities of DS-2969b, a novel GyrB inhibitor, against Clostridium difficile. Antimicrob Agents Chemother 62:e02157-17. doi:10.1128/AAC.02157-17. 29439962 PMC5913969

[27] Pulse M, Weiss W, Kers J, DeFusco A, Park J, Handfield M. 2019. Pharmacological, toxicological, and dose range assessment of OG716, a novel lantibiotic for the treatment of Clostridium difficile-associated infection. Antimicrob Agents Chemother 63:e01904-18. doi:10.1128/AAC.01904-18. 30670434 PMC6437525

[28] Swanson RN, Hardy DJ, Shipkowitz NL, Hanson CW, Ramer NC, Fernandes PB, Clement JJ. 1991. In vitro and in vivo evaluation of tiacumicins B and C against Clostridium difficile. Antimicrob Agents Chemother 35:1108–1111. doi:10.1128/aac.35.6.1108. 1929250 PMC284295

[29] Sattar A, Thommes P, Payne L, Warn P, Vickers RJ. 2015. SMT19969 for Clostridium difficile infection (CDI): in vivo efficacy compared with fidaxomicin and vancomycin in the hamster model of CDI. J Antimicrob Chemother 70:1757–1762. doi:10.1093/jac/dkv005. 25652749 PMC4498292

[30] Centers for Disease Control and Prevention. 2013. Antibiotic resistance threats in the United States, 2013. Centers for Disease Control and Prevention, Atlanta, GA.

[31] Navalkele BD, Chopra T. 2018. Bezlotoxumab: an emerging monoclonal antibody therapy for prevention of recurrent Clostridium difficile infection. Biologics 12:11–21. doi:10.2147/BTT.S127099. 29403263 PMC5779312

[32] Haber SL, Raney CRK, Larson TL, Lau JP. 2019. Fecal microbiota transplantation for recurrent Clostridioides difficile infection. Am J Health Syst Pharm 76:935–942. doi:10.1093/ajhp/zxz078. 31361890

[33] Andersson MI, MacGowan AP. 2003. Development of the quinolones. J Antimicrob Chemother 51 Suppl 1:1–11. doi:10.1093/jac/dkg212. 12702698

[34] Pham TDM, Ziora ZM, Blaskovich MAT. 2019. Quinolone antibiotics. Medchemcomm 10:1719–1739. doi:10.1039/c9md00120d. 31803393 PMC6836748

[35] Kumar M, Mathur T, Barman TK, Ramkumar G, Bhati A, Shukla G, Kalia V, Pandya M, Raj VS, Upadhyay DJ, Vaishnavi C, Chakrabarti A, Das B, Bhatnagar PK. 2012. In vitro and in vivo activities of the novel Ketolide RBx 14255 against Clostridium difficile. Antimicrob Agents Chemother 56:5986–5989. doi:10.1128/AAC.00015-12. 22869573 PMC3486553

[36] Levitus M, Rewane A, Perera TB. 2020. Vancomycin-resistant enterococci (VRE). StatPearls. StatPearls Publishing LLC, Treasure Island, FL. 30020605

[37] Sears P, Crook DW, Louie TJ, Miller MA, Weiss K. 2012. Fidaxomicin attains high fecal concentrations with minimal plasma concentrations following oral administration in patients with Clostridium difficile infection. Clin Infect Dis 55(Suppl 2):S116–S120. doi:10.1093/cid/cis337. 22752859 PMC3388019

[38] Deshpande A, Pasupuleti V, Thota P, Pant C, Rolston DD, Hernandez AV, Donskey CJ, Fraser TG. 2015. Risk factors for recurrent Clostridium difficile infection: a systematic review and meta-analysis. Infect Control Hosp Epidemiol 36:452–460. doi:10.1017/ice.2014.88. 25626326

[39] Gil F, Lagos-Moraga S, Calderón-Romero P, Pizarro-Guajardo M, Paredes-Sabja D. 2017. Updates on Clostridium difficile spore biology. Anaerobe 45:3–9. doi:10.1016/j.anaerobe.2017.02.018. 28254263

[40] Science Council of Japan. 2006. Guidelines for proper conduct of animal experiments. Science Council of Japan. http://www.scj.go.jp/en/animal/.

[41] Clinical and Laboratory Standards Institute. 2012. Methods for antimicrobial susceptibility testing of anaerobic bacteria; approved standard, 8th ed. CLSI document M11-A8. Clinical and Laboratory Standards Institute, Wayne, PA.

[42] Clinical and Laboratory Standards Institute. 2012. Performance standards for antimicrobial susceptibility testing; 22nd informational supplement. CLSI document M100-S22. Clinical and Laboratory Standards Institute, Wayne, PA.

[43] Fisher LM, Pan XS. 2008. Methods to assay inhibitors of DNA gyrase and topoisomerase IV activities. Methods Mol Med 142:11–23. doi:10.1007/978-1-59745-246-5_2. 18437302

[44] Vogelman BS, Craig WA. 1985. Postantibiotic effects. J Antimicrob Chemother 15(Suppl A):37–46. doi:10.1093/jac/15.suppl_a.37. 3980335

[45] Borenfreund E, Puerner JA. 1985. Toxicity determined in vitro by morphological alterations and neutral red absorption. Toxicol Lett 24:119–124. doi:10.1016/0378-4274(85)90046-3. 3983963

