# Supplementary material for: In Vitro and In Vivo Antibacterial Activities of a Novel Quinolone Compound, OPS-2071, against Clostridioides difficile
Source: Antimicrob Agents Chemother. 2021 Mar 18;65(4):e01170-20. doi: 10.1128/AAC.01170-20 (PMC8097418; doi:10.1128/AAC.01170-20)
Supplement: Supplemental file 1 [file AAC.01170-20-s0001.pdf]

Supplemental Material

TABLE S1. Antibacterial activity against 54 clinically isolated strains of *C. difficile*

CODE	OPS-2071	CPFX	LVFX	VCM	MTZ	FDX
1417-C-0005	0.031	16	8	2	2	0.063
1417-C-0006	0.063	32	8	2	>128	4
1417-C-0007	0.031	16	8	2	1	0.063
1417-C-0008	0.125	128	>128	2	2	0.031
1417-C-0009	0.125	128	>128	4	1	0.063
1417-C-0010	0.063	16	16	2	1	0.016
1417-C-0011	0.016	16	4	2	2	0.016
1417-C-0012	0.063	16	8	2	1	0.031
1417-C-0013	0.125	32	128	2	2	0.031
1417-C-0014	0.125	32	128	>128	>128	8
1417-C-0015	0.25	128	>128	2	2	0.063
1417-C-0016	0.25	128	>128	2	2	0.031
1417-C-0017	0.25	128	>128	4	1	0.063
1417-C-0018	0.031	16	8	2	1	0.063
1417-C-0019	0.5	32	32	2	2	0.063
1417-C-0020	0.016	16	4	2	1	0.063
1417-C-0021	0.125	128	>128	8	1	0.031
1417-C-0022	0.031	16	8	1	2	0.031
1417-C-0023	1	128	>128	4	1	0.031
1417-C-0024	0.063	32	128	1	2	0.125
1417-C-0025	0.5	128	>128	2	>128	16
1417-C-0026	0.25	128	>128	2	2	0.125
1417-C-0027	0.125	128	>128	2	1	0.031
1417-C-0028	0.063	16	8	>128	>128	>128
1417-C-0029	0.125	128	128	4	2	0.063
1417-C-0030	0.5	128	>128	1	2	0.031
1417-C-0031	0.063	32	128	1	2	0.063
1417-C-0032	0.125	128	>128	2	1	0.031
1417-C-0033	0.5	128	>128	1	2	0.063
1417-C-0034	0.063	32	128	1	2	0.063
1417-C-0035	0.5	128	>128	8	1	0.031
1417-C-0036	0.031	16	8	2	2	0.063
1417-C-0037	0.063	32	128	1	2	0.063
1417-C-0038	0.031	16	8	2	2	0.063
1417-C-0039	0.25	128	>128	32	1	>128
1417-C-0040	0.063	64	>128	2	1	0.031
1417-C-0041	0.063	128	128	4	>128	8
1417-C-0042	0.5	128	>128	4	2	0.063
1417-C-0043	0.063	16	4	2	2	0.031
1417-C-0044	0.031	16	4	2	2	0.063
1417-C-0050	0.125	128	>128	2	4	0.031
1417-C-0051	0.031	16	8	4	2	0.125
1417-C-0052	0.125	128	>128	2	1	0.016

1417-C-0053	0.5	128	>128	2	2	0.063
1417-C-0054	0.5	32	128	1	2	0.063
1417-C-0001	0.25	128	>128	2	2	0.031
1417-C-0002	0.063	32	128	1	2	0.063
1417-C-0003	0.031	16	16	2	2	8
1417-C-0004	0.063	32	128	1	2	0.125
1417-C-0050	0.5	128	>128	1	2	0.063
1417-C-0051	0.063	16	8	2	2	0.063
1417-C-0052	1	128	>128	2	2	0.063
1417-C-0053	0.25	128	>128	2	2	0.031
1417-C-0054	0.031	16	4	2	2	0.063

CODE: Internal identification number of microorganisms.

CPFX: ciprofloxacin, LVFX: levofloxacin, VCM: vancomycin, MTZ: metronidazole, FDX: fidaxomicin

TABLE S2. Antibacterial activity against 20 hypervirulent strains of *C. difficile*

CODE	RIBOTYPE
1471-C-0070	78
1471-C-0058	78
1471-C-0069	78
1471-T-0026	18
1471-T-0025	18
1471-T-0010	23
1471-T-0024	23
1471-T-0012	27
1471-T-0028	27
1471-T-0027	27
1471-T-0011	27
1471-T-0032	56
1471-T-0031	56
1471-T-0033	78
1471-T-0018	78
1471-T-0034	78
1471-T-0017	78
1471-T-0037	244
1471-T-0035	244
1471-T-0036	244

CODE: Internal identification number of microorganisms.

TABLE S3. Effect of pH on antibacterial activity against *C. difficile* ATCC 700057

		MIC (µg/mL)		
		OPS-2071	Vancomycin	Fidaxomicin
pH	6	0.016	2	0.03
	7	0.016	2	0.06
	8	0.03	2	0.06

MICs for *C. difficile* ATCC 700057 were determined visually by the agar dilution method. In order to examine the effect of pH, Brucella broth supplemented with 5 µg/mL hemin and 1 µg/mL vitamin K1 was adjusted using 1N NaOH or 1N HCl and added agar powder (final concentration 1.5%) to produce pH values of 6, 7, or 8. The differences in the MICs of OPS-2071 over this pH range were comparable to those of vancomycin and fidaxomicin.
